# Retinal microvascular parameters are not associated with diabetes in the Northern Ireland Cohort for the Longitudinal Study of Ageing

**DOI:** 10.1007/s11845-021-02704-1

**Published:** 2021-07-09

**Authors:** Rachael Ann O’Neill, Alexander Peter Maxwell, Frank Kee, Ian Young, Bernadette McGuinness, Ruth E. Hogg, Sharon Cruise, Gareth J. McKay

**Affiliations:** Centre for Public Health, Queens University Belfast, Belfast, Ireland

**Keywords:** Diabetes, HbA1c, Retinal microvascular parameters

## Abstract

**Background:**

The retinal microvasculature offers unique non-invasive evaluation of systemic microvascular abnormalities. Previous studies reported associations between retinal microvascular parameters (RMPs) and diabetes. The aim of this study was to assess associations between RMPs and diabetes in a cross-sectional analysis of older persons from the Northern Ireland Cohort for the Longitudinal Study of Ageing (NICOLA).

**Methods:**

RMPs (central retinal arteriolar/venular equivalents, arteriolar to venular ratio, fractal dimension, and tortuosity) were measured from optic disc-centred fundus images using semi-automated software. Associations were assessed between RMPs and diabetes status with adjustment for potential confounders.

**Results:**

Data were included for 1762 participants with 209 classified as having diabetes. Participants had a mean age of 62.1 ± 8.5 years, and 54% were female. As expected, participants with diabetes had significantly higher mean glycated haemoglobin A1c compared to participants without diabetes (57.4 ± 17.6 mmol/mol versus 37.0 ± 4.2 mmol/mol, respectively). In unadjusted and minimally adjusted regression, arteriolar to venular ratio, arteriolar tortuosity and venular tortuosity were significantly associated with diabetes (minimally adjusted odds ratio [OR] = 0.85; 95% confidence intervals [CIs] 0.73, 0.99; *P* = 0.04, OR = 1.18; 95% CI 1.02, 1.37; *P* = 0.03 and OR = 1.20; 95% CI 1.04, 1.38; *P* = 0.01, respectively), although all failed to remain significant following adjustment for potential confounders. No additional associations between other RMPs and diabetes were detected.

**Conclusion:**

Despite previously reported associations between diabetes and RMPs, our study failed to corroborate these associations in an older community-based cohort.

**Supplementary information:**

The online version contains supplementary material available at 10.1007/s11845-021-02704-1.

## Background

Global life expectancy is increasing and in Northern Ireland, the most rapidly growing demographic group are those > 50 years, highlighting the importance of research on age-related conditions [1]. Diabetes is imposing an increasing burden on public health systems, and older individuals are more likely to be over-represented among those with diabetes [2, 3]. Measurement of glycated haemoglobin A1c (HbA1c) is a diagnostic test that reflects the average blood sugar level over the previous 2–3 months, enabling effective monitoring of intervention strategies to lower blood sugar levels and associated risk of diabetic complications [4, 5]. The pathogenesis of diabetes is complex leading to microvascular dysfunction [6, 7], following prolonged periods of chronic hyperglycaemia that commonly leads to damage to the kidneys, retinas and peripheral nervous system and increased cardiovascular risk [8]. The retinal microvasculature comprises a collection of small arterioles and venules. Their examination provides a unique and non-invasive opportunity to evaluate systemic microvasculature variation and abnormalities that may reflect similar changes elsewhere within the microvascular system [3, 9, 10].

Advances in retinal imaging software and analysis applications can provide for opportunistic identification of microangiopathic variation in the eye that may reflect similar microvascular anomalies elsewhere in the body [11, 12]. Additionally, variation in retinal microvascular parameters (RMPs) may reflect aetiological mechanisms that are characteristic of diabetes pathogenesis such as oxidative stress, endothelial dysfunction, inflammation and hypertension [13–17]. A recent systematic review and meta-analysis of several population-based studies reported that increased retinal venular dilation, but not retinal arteriolar narrowing, was significantly associated with an increased risk for diabetes [3]. Nevertheless, although most prospective studies reported associations between retinal venular dilation and diabetes, these data have not always been consistent with cross-sectional analyses [3, 18–23]. Other RMPs are reported to be associated with diabetes. Increased retinal arteriolar [24] and venular tortuosity [24, 25] were reported to be significantly increased in individuals with type 2 diabetes or elevated HbA1c. Yau et al. reported increased fractal dimensions associated with impaired glucose tolerance and/or type 2 diabetes [26]. In contrast, Broe et al. found that decreased fractal dimensions were associated with greater risk of complications in type 1 diabetes [27]. As such, the aim of this study was to assess RMPs in association with measures of HbA1c and diabetes status in a cross-sectional analysis of older persons from the Northern Ireland Cohort for the Longitudinal Study of Ageing (NICOLA).

## Methods

### Study population

NICOLA is a longitudinal cohort study of 8468 community dwelling men and women aged 50 years and over, resident in Northern Ireland (individuals in care homes or other residential institutions were excluded at baseline) [28]. The study, established in 2013, has three main components: a computer-aided personal interview (CAPI), a self-completion questionnaire and health assessment. The CAPI was extensive in scope and included assessment of demographic, social and health-related factors. Measures of cardiovascular, physical, cognitive and visual function were determined; biological samples were collected and a visual health assessment undertaken that included retinal fundus photography. Written informed consent was obtained from participants prior to taking part, following ethical approval from the School of Medicine, Dentistry and Biomedical Sciences Ethics Committee, Queen’s University Belfast (SREC 12/23) and in accordance with the Helsinki Declaration.

### Measurement of retinal images

**Fig. 1:**
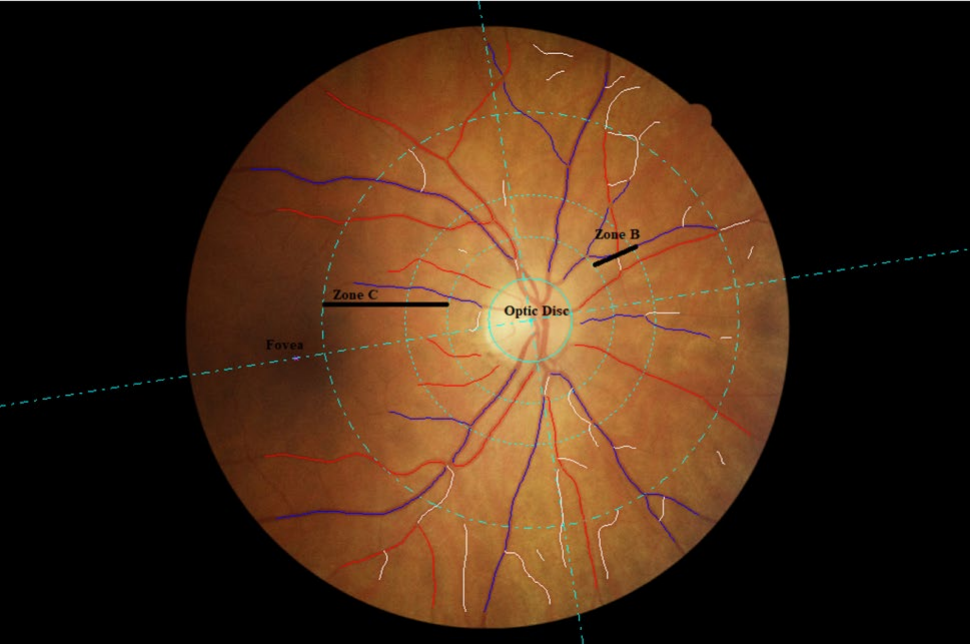
Retinal fundus image assessed using the VAMPIRE software. Optic disc–centred retinal fundus image assessed using the Vessel Assessment and Measurement Platform for Images of the Retina (VAMPIRE) software. Arterioles (red), venules (blue) and deleted segments (white) are indicated. The retinal microvascular parameters for arteriolar and venular calibre (CRAE, CRVE and AVR) are calculated from measurements captured in zones B (1.0 to 1.5 optic disc diameters from the centre of the optic disc). Fractal dimension and tortuosity are calculated from measurements captured in zone C (1.0 to 2.5 optic disc diameters from the centre of the optic disc)

Retinal photography was performed following dilation from a single drop of 1% tropicamide using a Canon CX-1 Digital Fundus Camera (Canon USA, Melville, NY, USA). RMPs (central retinal arteriolar equivalent [CRAE], central retinal venular equivalents [CRVE], arteriolar to venular ratio [AVR], fractal dimension and tortuosity) were measured from optic disc–centred fundus images and analysis undertaken using the semi-automated software Vessel Assessment and Measurement Platform for Images of the Retina (VAMPIRE; VAMPIRE group, Universities of Dundee and Edinburgh, Scotland, Version 3.1; Fig. 1), by a trained grader, blinded to participant data [29, 30]. Analysis was undertaken on images taken from the left eye except when unavailable or of insufficient quality, in which case the right eye image was used. A paired samples t-test compared a sub-sample of left and right eye measurements. Intraclass correlation coefficients (ICCs) were calculated to assess intergrader reliability with mean values of 0.87 (CRAE) and 0.91 (CRVE).

### Measurement and classification of diabetic status

**Fig. 2:**
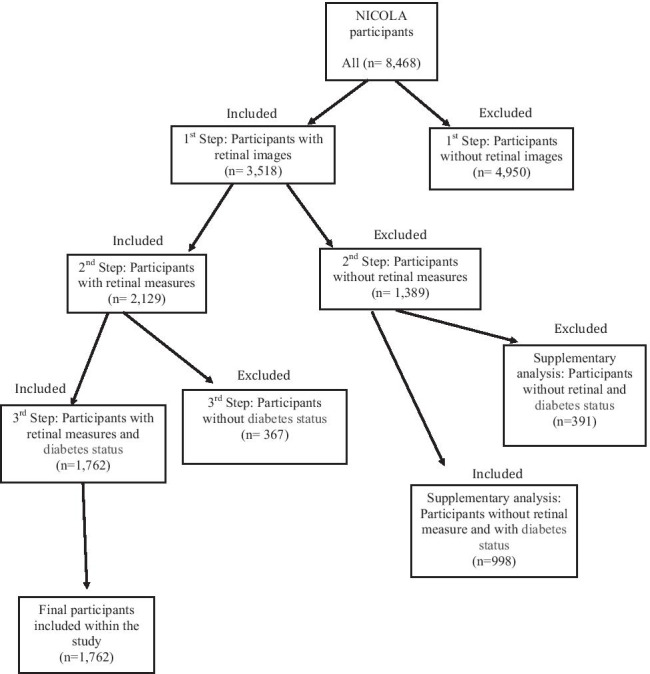
A flow chart of participant inclusion and exclusion criteria

Diabetes status was categorised on the basis of self-reported diabetes at CAPI, use of diabetic medications or HbA1c ≥ 48 mmol/mol. Participants were excluded if diabetes status or retinal images were missing or of insufficient quality for analysis (Fig. 2).

### Other variables

Mean arterial blood pressure (MABP) was calculated as the average of two individual systolic blood pressure (SBP) and diastolic blood pressure (DBP) measurements (2/3 DBP + 1/3 SBP). Smoking status was categorised as current smokers versus non-smokers. Alcohol consumption was categorised: non-drinker, light drinker (1–7 units per week), moderate to heavy drinker (> 7 units per week). Cardiovascular disease (CVD) was characterised by self-report on a history of angina, heart attack, congestive heart failure or stroke. Participant height was measured to the nearest centimetre using a seca 240 wall-mounted measuring rod, and weight was measured in kilograms using seca electronic floor scales. Triglycerides and high- and low-density lipoprotein (HDL and LDL) were measured from individual participant blood samples. Physical activity (PA) was categorised as inactive, low, moderate or highly active in accordance with the Global Physical Activity Questionnaire (GPAQ), scored by calculating the average time per day spent in each activity domain (work, transport and leisure) and the intensity of PA during that time [31, 32].

### Statistical analysis

All analyses were performed using Statistical Package for Social Sciences (Version 24.0. Armonk, NY: IBM Corp). Before inclusion in regression models, all RMPs were transformed into standardised Z-scores (a standard deviation [SD] increase from the mean). Population characteristics were summarised as mean and SD for continuous variables or frequencies and percentages for categorical variables. Between-group differences were assessed using chi-squared tests to compare categorical variables for demographic and clinical factors and t-tests for continuous variables according to diabetes status. Binary logistic regression was used to evaluate associations between RMPs and diabetic status. Additional sensitivity analyses included excluding control participants with pre-diabetes (HbA1c values between 42 and 47 mmol/mol) and linear regression modelling to evaluate associations between RMPs and HbA1c. Minimally adjusted regression models included age and sex, while fully adjusted models also included smoking status, CVD, alcohol consumption, MABP, physical activity level, BMI, triglycerides and HDL and LDL. P < 0.05 was considered statistically significant.

## Results

Data for retinal imaging of sufficient quality and HbA1c was available for 1762 participants who attended the health assessment; 209 were classified as diabetic (12%). Table 1 provides study participant summary characteristics. Participants had a mean age of 62.1 ± 8.5 years, and 54% were female. Mean HbA1c was 39.4 ± 9.8 mmol/mol, MABP was 98.0 ± 12.3 mm Hg, and 27% were characterised by high levels of physical activity. As expected, participants with diabetes had significantly higher mean HbA1c (57.4 mmol/mol versus 37.0 mmol/mol, respectively), lower estimated glomerular filtration rate (77.4 ml/min/1.73 m^2^ versus 82.8 ml/min/1.73m^2^), were hypertensive (43.1% versus 35.6%; defined as a mean systolic blood pressure ≥ 140 mm Hg or mean diastolic blood pressure ≥ 90 mm Hg) and tended to be older (65.1 ± 9.0 years versus 61.7 ± 8.4 years) than those without diabetes (Table 1). They also had higher mean triglycerides (1.9 ± 1.2 mmol/L versus 1.6 ± 0.9 mmol/L, respectively) but lower mean HDL and LDL cholesterol (HDL 1.4 ± 0.4 mmol/L versus 1.7 ± 0.5 mmol/L and LDL 2.8 ± 1.1 mmol/L versus 3.5 ± 1.1 mmol/L). A higher percentage of participants with diabetes were more likely to refrain from alcohol consumption (34% versus 21%), have a higher BMI (31.4 ± 5.7 kg/m^2^ versus 28.2 ± 4.7 kg/m^2^, respectively), have a history of CVD (19% versus 6%) and be less physically active (12% versus 29%; Table 1). Participants with diabetes were also more likely to be taking lipid-modifying agents (65.1% versus 23.2%) and using antihypertensive medication (3.8% versus 1.5%). Only a small number of participants had type 1 diabetes (*n* = 3), had self-reported diabetic retinopathy (*n* = 9) or were taking prescribed medication as a treatment for diabetes (*n* = 85). Comparisons of participant characteristics between those with good-quality retinal imaging included in the study and those excluded due to poor-quality retinal imaging are presented in Supplementary Table 1.

**Table 1:** Participant summary characteristics

Participant characteristics	All (*n* = 1762)	No diabetes (*n* = 1553)	Diabetes (*n* = 209)	*P*-value
Mean age (years, SD)	62.1 ± 8.5	61.7 ± 8.4	65.1 ± 9.0	< 0.01
Female, *n* (%)	944 (53.6)	841 (54.2)	103 (49.3)	0.19
Smoking status, yes *n* (%)	171 (9.7)	149 (9.6)	22 (10.5)	0.67
Alcohol consumption, non-drinker, *n* (%)	393 (22.3)	323 (20.8)	70 (33.5)	< 0.01
Physical activity level, highly active, *n* (%)	481 (27.3)	455 (29.3)	26 (12.4)	< 0.01
Cardiovascular disease, yes *n* (%)	134 (7.6)	94 (6.1)	40 (19.1)	< 0.01
Hypertension, *n* (%)	643 (36.5)	553 (35.6)	90 (43.1)	0.04
Mean eGFR (ml/min/1.73m^2^)	82.2 (14.8)	82.8 (14.4)	77.4 (17.3)	< 0.01
Mean BMI (kg/m^2^, SD)	28.6 ± 4.9	28.2 ± 4.7	31.4 ± 5.7	< 0.01
Mean arterial blood pressure (mm Hg, SD)	98.0 ± 12.3	97.8 ± 12.4	99.6 ± 11.5	0.05
Mean triglyceride (mmol/L, SD)	1.7 ± 0.9	1.6 ± 0.9	1.9 ± 1.2	< 0.01
Mean HDL cholesterol (mmol/L, SD)	1.6 ± 0.5	1.7 ± 0.5	1.4 ± 0.4	< 0.01
Mean LDL cholesterol (mmol/L, SD)	3.4 ± 1.1	3.5 ± 1.1	2.8 ± 1.1	< 0.01
Mean HbA1c (mmol/mol, SD)	39.4 ± 9.8	37.0 ± 4.2	57.4 ± 17.6	< 0.01
Using lipid modifying agents, *n* (%)	497 (28.2)	361 (23.2)	136 (65.1)	< 0.01
Using antihypertensive drugs, *n* (%)	31 (1.8)	23 (1.5)	8 (3.8)	0.02

Values are *n* (%) for categorical variables and mean ± SD for continuous variables. *P* values were calculated by independent samples t and chi-squared tests. Hypertension was defined as a systolic blood pressure ≥ 140 mm Hg or a diastolic blood pressure ≥ 90 mm Hg

*eGFR* estimated glomerular filtration rate, *HbA1c* glycated haemoglobin, *BMI* body mass index, *HDL* high-density lipoprotein, *LDL* low-density lipoprotein, *SD* standard deviation

Comparisons of CRAE and CRVE from left and right eye images from 75 participants were not significantly different (PCrae = 0.08; PCrve = 0.89). Table 2 summarises mean RMPs. Both mean arterial and venular tortuosity were significantly different between those with and without diabetes (*P* < 0.05; Table 2).

**Table 2:** Summary of participant retinal microvascular parameters

Retinal microvascular parameters	All (*n* = 1762)	Non-diabetic (*n* = 1553)	Diabetic (*n* = 209)	*P*-value
Mean CRAE (PX, SD)	29.655 ± 2.196	29.670 ± 2.204	29.545 ± 2.139	0.44
Mean CRVE (PX, SD)	40.898 ± 3.275	40.854 ± 3.254	41.230 ± 3.423	0.12
Mean AVR (SD)	0.728 ± 0.062	0.729 ± 0.062	0.720 ± 0.061	0.04
Mean fractal dimension arteriolar (SD)	1.558 ± 0.051	1.557 ± 0.051	1.561 ± 0.053	0.36
Mean fractal dimension venular (SD)	1.540 ± 0.050	1.540 ± 0.050	1.541 ± 0.053	0.92
Mean tortuosity arteriolar (SD)^a^	0.111 ± 0.148	0.108 ± 0.138	0.133 ± 0.208	0.02
Mean tortuosity venular (SD)^a^	0.068 ± 0.105	0.066 ± 0.099	0.083 ± 0.140	0.01

Values are mean ± SD for continuous variables. *P* values were calculated by independent samples t tests

*CRAE* central retinal arteriolar equivalent, *CRVE* central retinal venular equivalent, *AVR* retinal arteriolar/venular ratio, *SD* standard deviation, *PX* pixels

^a^Tortuosity values were multiplied by 1000 in order to be shown in table. *P* < 0.05 was considered statistically significant

In unadjusted and minimally adjusted regression, AVR, arteriolar tortuosity and venular tortuosity were significantly associated with diabetes (minimally adjusted odds ratio [OR] = 0.85; 95% confidence intervals [CI] 0.73, 0.99; *P* = 0.04, OR = 1.18; 95% CI 1.02, 1.37; *P* = 0.03 and OR = 1.20; 95% CI 1.04, 1.38; *P* = 0.01, respectively), although these did not remain significant following adjustment for the potential confounding effects of smoking status, alcohol consumption, CVD, MABP, PA, BMI, triglycerides, HDL and LDL (OR = 0.88; 95% CI 0.75, 1.04; *P* = 0.13, OR = 1.13; 95% CI 0.96, 1.32; *P* = 0.13 and OR = 1.14; 95% CI 0.97, 1.32; *P* = 0.11, respectively; Table 3). No additional associations between diabetes and RMPs were detected. A sensitivity analysis that excluded 224 control participants with a HbA1c level between 42 and 47 mmol/mol did not differ significantly from the findings of the primary analysis (data not shown). In a sensitivity linear regression analysis of associations between RMPs and HbA1c, only arteriolar tortuosity was significantly associated with HbA1c in both unadjusted and minimally adjusted models (β = 0.006; 0.001, 0.010; *P* = 0.02), but this did not remain significant in the fully adjusted model (Supplementary Table 2).

**Table 3:** Logistic regression analysis of retinal microvascular parameters and diabetic status

	Minimally adjusted				Fully adjusted		
Retinal parameter	OR	95% CI	*P*-value	OR		95% CI	*P*-value
CRAE (PX)^a^	0.92	0.79, 1.07	0.28	0.97		0.82, 1.14	0.67
CRVE (PX)^a^	1.10	0.95, 1.27	0.19	1.11		0.95, 1.29	0.21
AVR^a^	0.85	0.73, 0.99	0.04	0.88		0.75, 1.04	0.13
Fractal dimension arteriolar^a^	1.10	0.95, 1.28	0.21	1.15		0.98, 1.36	0.09
Fractal dimension venular^a^	1.04	0.89, 1.21	0.64	1.02		0.87, 1.21	0.77
Tortuosity arteriolar^a,b^	1.18	1.02, 1.37	0.03	1.13		0.96, 1.32	0.13
Tortuosity venular^a,b^	1.20	1.04, 1.38	0.01	1.14		0.97, 1.32	0.11

*CRAE* central retinal arteriolar equivalent, *CRVE* central retinal venular equivalent, *AVR* retinal arteriolar/venular ratio, *CI* confidence interval, *OR* odds ratio, *PX* pixels

^a^RMPs were transformed into standardised Z-scores before inclusion in regression models

^b^Tortuosity values were log-transformed before inclusion in regression models to produce normal distribution. Minimally adjusted models included age and sex, with fully adjusted models also including smoking status, alcohol consumption, CVD, MABP and physical activity level, body mass index, triglycerides and high- and low-density lipoprotein levels

## Discussion

Advances in retinal imaging technologies have enabled improved quantitative assessment of RMPs as surrogates of microvascular health [33]. Several studies have reported associations between RMPs and diabetes, but the findings between longitudinal and cross-sectional studies have not always been consistent, in part due to variation in the reported unit measures included in statistical analyses (e.g. pixels, microns, standardised Z-scores and per standard deviation increase) and the availability of potential confounding variables considered in adjusted models [18–27, 34–39]. In this cross-sectional analysis of 1762 older persons from the NICOLA study categorised as either diabetic or non-diabetic, we found that AVR and arteriolar and venular tortuosity were significantly associated with diabetes when adjusted for age and sex (Table 3), but none remained significantly associated with diabetes following adjustment for multiple potential confounders. Other similar population-based studies, such as the Rotterdam Study, also failed to detect significant associations between RMPs and diabetes status [38]. In sensitivity analyses that excluded those with pre-diabetes and evaluated associations between RMPs and HbA1c, similar findings to the primary analysis presented were also identified. A recent systematic review and meta-analysis reported retinal venular widening in association with an increased risk of incident diabetes [3]. Kifley et al. also reported wider retinal arteriolar calibre in association with diabetes [19, 37], although this finding contrasted with the retinal arteriolar narrowing reported by Wong and colleagues [34, 35]. Jegnathan and colleagues reported wider retinal arteriolar and venular calibre in association with diabetes and increasing glucose levels [18]. Although our findings suggested retinal arteriolar narrowing and venular dilation was associated with diabetes, these associations did not remain significant following adjustment for potential confounders. Nevertheless, given the meta-analysis performed by Sabanayagam and colleagues had more than ten times the number of diabetes cases, power to detect associations is likely to have been much reduced in our study [3].

Experimental studies have implicated diabetes-induced microvascular modification and structural morphological changes in retinal vessel complexity and density defined by fractal dimension [40]. Variation in retinal fractal dimension may be increased under hyperglycaemic conditions, leaving the microvasculature susceptible to further damage from additional vascular risk factors [35]. Few studies have evaluated associations between retinal fractal dimension and tortuosity with diabetic status [24–27]. Broe et al. found retinal vascular fractal dimension to be associated with microvascular dysfunction in diabetes that may be indicative of common pathogenic pathways, making it a possible means of stratifying diabetic risk [27]. Yau and colleagues also evaluated associations between retinal fractal dimension and diabetes. They reported that increased fractal dimension was significantly associated with participants with diabetes, but not in those with impaired glucose metabolism [26]. Sasongko et al. reported retinal arteriolar and venular tortuosity were both significantly increased in participants with diabetes, without the presence of diabetic retinopathy. They concluded that individuals with diabetes had more tortuous retinal vessels than controls without diabetes [24]. Owen and colleagues showed a graded association with both tortuosity and width of retinal venules and metabolic risk factors such as HbA1c, even among people without clinical diabetes [25]. The directions of effect in those with diabetes were also similar to those reported in our study for both, arteriolar and venular tortuosity in unadjusted and minimally adjusted models, despite not remaining significant in the fully adjusted models.

There were several limitations to our study. Firstly, although we adjusted for potential confounders, there is still the possibility of residual confounding. Secondly, the mainly Caucasian study population aged greater than 50 years may be a limitation to the generalisability of any findings to other populations of different ages and ethnicities. Finally, variation in retinal imaging software based on different algorithms may make comparisons between studies challenging [3].

Despite these limitations, our study had several strengths including the population-based design. The NICOLA study included well-characterised participants that captured a broad range of demographic factors and clinical variables including comorbidities and medications used that enable comprehensive characterisation of diabetic status. In addition, the availability of optic disc-centred retinal fundus images provides improved sensitivity for the quantification of RMPs compared to macula-centred images, which are largely restricted to the retinal temporal arcades. Although assessment of retinal images was based on a single eye, previous studies have reported a high correlation of RMPs with the fellow eye, supported by our sensitivity analysis of comparisons of vessel diameters in 75 participants [41–43].

In conclusion, although previous studies have reported contrasting associations between diabetes and RMPs, we failed to detect any significant associations in this older community-based cohort. The direction of effect that we observed supported those from a previous large meta-analysis but consistency around confounding variables, sample size and variation in algorithm-based measurement increases the challenges of cross-study comparisons.

## Supplementary information

Below is the link to the electronic supplementary material.

Supplementary file1 (DOCX 18 KB)

## Data Availability

The data that support the findings of this study are available from the Northern Ireland Cohort for Longitudinal Ageing (NICOLA), but restrictions apply to the availability of this data. Data access is available by request through the NICOLA Data Access Committee.

## Abbreviations

NICOLA: The Northern Ireland Cohort for the Longitudinal Study of Ageing
BMI: Body mass index
HDL: High-density lipoprotein
LDL: Low-density lipoprotein
CRAE: Central retinal arteriolar equivalent
CRVE: Central retinal venular equivalent
AVR: Retinal arteriolar/venular ratio
SD: Standard deviation
PX: Pixels
CPH: Centre for Public Health
CVD: Cardiovascular disease
SBP: Systolic blood pressure
DBP: Diastolic blood pressure
CI: Confidence intervals
β: Beta value
μm: Microns
ICCs: Intraclass correlation coefficients
VAMPIRE: Vessel Assessment and Measurement Platform for Images of the Retina
MABP: Mean arteriole blood pressure
RMPs: Retinal microvascular parameters
Yrs: Years
HbA1c: Glycated haemoglobin
