# Supplementary material for: Retinal microvascular parameters are not associated with diabetes in the Northern Ireland Cohort for the Longitudinal Study of Ageing
Source: Ir J Med Sci. 2021 Jul 9;191(3):1209–15. doi: 10.1007/s11845-021-02704-1 (PMC9135822; doi:10.1007/s11845-021-02704-1)
Supplement: Supplementary file 1 — Supplementary file1 (DOCX 18 KB) [file 11845_2021_2704_MOESM1_ESM.docx]

**Supplementary Table 1**: Comparison of demographic characteristics between all participants with retinal fundus imaging with and without VAMPIRE retinal measures.

| **Participant characteristics** | **Participants with images included (n=1,762)** | **Participants with images excluded (n=998)** | **P- value** |
| --- | --- | --- | --- |
| Mean age (years, SD) | 62.1 ± 8.5 | 66.5 ± 9.2 | <0.01 |
| Female, n (%) | 944 (53.5) | 489 (49.0) | 0.02 |
| Smoking Status, yes n (%) | 171 (9.7) | 106 (10.6) | 0.44 |
| Alcohol consumption, non-drinker, n (%) | 393 (22.3) | 263 (26.4) | 0.05 |
| Cardiovascular disease, yes n (%) | 134 (7.6) | 110 (11.0) | <0.01 |
| Mean BMI (kg/m^2^, SD) | 28.6 ± 4.9 | 28.9 ± 4.9 | 0.19 |
| Mean arterial blood pressure (mmHg, SD) | 98.0 ± 12.3 | 99.4 ± 12.5 | <0.01 |
| Mean triglyceride (mmol/L, SD) | 1.7 ± 0.9 | 1.6 ± 0.9 | 0.18 |
| Mean HDL cholesterol (mmol/L, SD) | 1.6 ± 0.5 | 1.6 ± 0.4 | 0.04 |
| Mean LDL cholesterol (mmol/L, SD) | 3.4 ± 1.1 | 3.2 ± 1.1 | <0.01 |
| Mean HbA1c (mmol/mol, SD) | 39.4 ± 9.8 | 40.2 ± 8.9 | 0.04 |
| Diabetes, yes n (%) | 209 (11.9) | 151 (15.1) | 0.01 |
| Diabetes, no n (%) | 1553 (88.1) | 847 (84.7) | 0.01 |

Values are n (%) for categorical variables and mean ± SD for continuous variables. P values were calculated by independent samples t and chi squared tests. Abbreviations: HbA1c, glycated haemoglobin; BMI, body mass index; HDL, high-density lipoprotein; LDL, low-density lipoprotein; SD, standard deviation. P < 0.05 was considered statistically significant.

**Supplementary Table 2**. Linear regression analysis of retinal microvascular parameters and HbA1c.

|  |  | **Unadjusted** |  | **Minimally Adjusted** | | | | | **Fully Adjusted** | |
| --- | --- | --- | --- | --- | --- | --- | --- | --- | --- | --- |
| **Retinal parameter** | **B** | **95% CI** | **P value** | **B** | **95% CI** | **P value** | **B** | **95% CI** | | **P value** |
| ^a^CRAE (PX) | -0.001 | -0.006, 0.004 | 0.70 | -0.002 | -0.007, 0.003 | 0.49 | 0.001 | -0.005, 0.005 | | 0.92 |
| ^a^CRVE (PX) | 0.002 | -0.003, 0.007 | 0.42 | 0.001 | -0.004, 0.006 | 0.62 | 0.001 | -0.004, 0.006 | | 0.57 |
| ^a^AVR | -0.003 | -0.007, 0.002 | 0.27 | -0.003 | -0.007, 0.002 | 0.27 | -0.002 | -0.007, 0.003 | | 0.52 |
| ^a^Fractal dimension arteriolar | 0.001 | -0.003, 0.006 | 0.60 | 0.002 | -0.002, 0.007 | 0.33 | 0.003 | -0.002, 0.008 | | 0.18 |
| ^a^Fractal dimension venular | -0.003 | -0.007, 0.002 | 0.30 | -0.001 | -0.006, 0.003 | 0.55 | -0.002 | -0.007, 0.003 | | 0.53 |
| ^ab^Tortuosity arteriolar | 0.006 | 0.001, 0.011 | 0.02 | 0.006 | 0.001, 0.010 | 0.02 | 0.004 | -0.001, 0.009 | | 0.09 |
| ^ab^Tortuosity venular | 0.004 | -0.001, 0.009 | 0.08 | 0.004 | -0.001, 0.009 | 0.11 | 0.002 | -0.003, 0.007 | | 0.45 |
|  |  |  |  |  |  |  |  |  | |  |

Abbreviations: CRAE, central retinal arteriolar equivalent; CRVE, central retinal venular equivalent; AVR, retinal arteriolar/venular ratio; CI, confidence interval; OR, odds ratio; PX, pixels. ^a^RMPs were transformed into standardised Z-scores before inclusion in regression models. ^b^Tortuosity values were log-transformed before inclusion in regression models to produce normal distribution. Minimally adjusted models included age and sex, with fully adjusted models also including smoking status, alcohol consumption, CVD, MABP and physical activity level, body mass index, triglycerides and high and low-density lipoprotein levels.
